# A 12-month structured exercise reduces epicardial adipose volume in a frequency-dependent manner and lowers serum M-CSF in community-dwelling older adults: a randomized controlled trial

**DOI:** 10.3389/fpubh.2026.1938784

**Published:** 2026-09-16

**Authors:** Xiaojing Xue, Simiao Chen, Yanling Cheng, Qiaowei Li, Zhiqiang Ye, Haowen Li, Feng Huang, Fang Wang

**Affiliations:** 1 Shengli Clinical Medical College of Fujian Medical University, Fuzhou, China; 2 Institute of Clinical Geriatrics, Fuzhou University Affiliated Provincial Hospital, Fuzhou, China; 3 Department of Neurology, Fuzhou University Affiliated Provincial Hospital, Fuzhou, China; 4 Fujian Key Laboratory of Geriatrics, Fuzhou, China; 5 School of Nursing, Fujian University of Traditional Chinese Medicine, Fuzhou, China

**Keywords:** epicardial adipose tissue, epicardial adipose volume, inflammation, macrophage colony-stimulating factor, older adults, randomized controlled trial, structured exercise intervention

## Abstract

**Objective:**

Exercise is a well-established strategy for promoting healthy aging and reducing cardiometabolic risk; however, its effects on epicardial adipose volume (EAV) and associated inflammatory pathways in older adults remain insufficiently understood. This randomized controlled trial examined whether a 12-month structured exercise program reduces EAV in community-dwelling older adults, and secondarily explored changes in serum pro-inflammatory cytokines.

**Methods:**

This single-center pragmatic randomized controlled trial incorporated a Zelen design. A total of 156 older adults (≥60 years) were evenly allocated to a structured exercise (STE) group or a control group. The control group received health education only; the STE group performed one supervised center-based session plus at least two home-based sessions per week, with matched intensity and duration. EAV, body fat parameters, and circulating pro-inflammatory factors were measured at baseline and after 12 months.

**Results:**

Among the randomized participants, 57 individuals in the control group and 61 individuals in the STE group completed the 12-month follow-up (55 men, 46.6%; mean age 69.17 ± 4.41 years). The absolute EAV change differed significantly between groups (STE vs. control: −15.15 mL, 95% CI –22.61 to −7.69, *p* < 0.0001). After adjusting for baseline values, only serum macrophage colony-stimulating factor (M-CSF) showed a significant between-group difference (−1.75 pg/mL, 95% CI –3.34 to −0.16, *p* = 0.032). Univariate linear regression indicated that each additional weekly exercise session was associated with a 3.156 mL greater EAV reduction (*R*^2^ = 0.084).

**Conclusion:**

A 12-month structured exercise intervention was associated with reduced EAV and lower circulating M-CSF levels in community-dwelling older adults, with greater reductions observed among participants engaging in more frequent weekly exercise sessions. These findings support regular supervised exercise as a non-pharmacological strategy to reduce cardiac adiposity and modulate inflammation in aging populations.

**Clinical trial registration:**

https://www.chictr.org.cn/indexEN.html, ChiCTR2400081824.

## Graphical abstract

**Figure:**
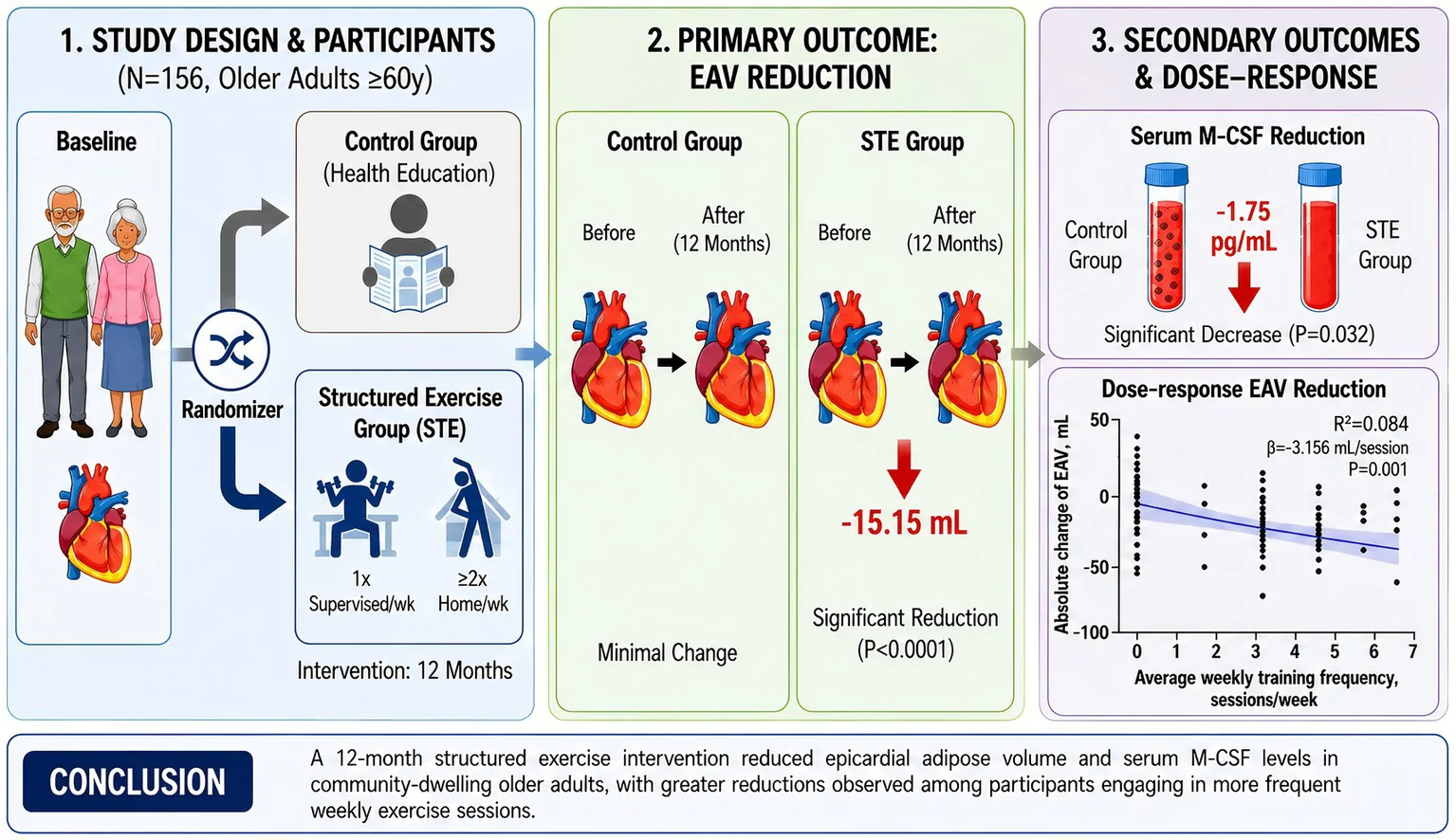

## 1 Introduction

The global population is aging rapidly, creating substantial public health challenges related to the prevention and management of age-associated chronic diseases. Advancing age is accompanied by progressive alterations in body composition, including increased total adiposity, visceral fat accumulation, and redistribution of fat depots toward ectopic locations (1). These changes contribute to metabolic dysfunction, insulin resistance, chronic low-grade inflammation, and elevated cardiovascular risk among older adults (2). Epicardial adipose tissue (EAT) – a unique ectopic fat depot located between the myocardium and the visceral pericardium – has drawn growing interest because it can influence cardiac function and coronary vessels through mechanical, endocrine, and paracrine pathways (3). Aging is a major determinant of EAT expansion. Abbara et al. (4) reported that individuals over 65 years have on average 22% higher EAT volume than younger adults. Karadag et al. (5) found a significant positive association between age and EAT after adjusting for confounders. Similarly, a cross-sectional study of 880 participants aged 45–74 years showed that the proportion with echocardiographic EAT thickness >5 mm increased significantly with age (6). This age-related trend reflects both general adipose expansion and a propensity for ectopic fat deposition.

Visceral adiposity fuels systemic inflammation and elevates cardiovascular risk in older adults. EAT is metabolically active and exhibits a pro-inflammatory, pro-fibrotic, and pro-atherogenic phenotype as its volume increases, with greater immune cell infiltration and elevated secretion of inflammatory adipokines (7). EAT has been proposed as an imaging biomarker for cardiovascular risk stratification (8, 9). Thresholds of 113–120 cm^3^ have been suggested to predict future cardiovascular events (10, 11). A meta-analysis of 29 cohort studies (19,709 participants) showed that increased EAT thickness or volume was significantly associated with cardiac death (OR = 2.53), myocardial infarction (OR = 2.63), coronary revascularization (OR = 2.99), and atrial fibrillation (OR = 4.04) (12).

Previous work has demonstrated that exercise improves multiple drivers of healthy aging, including cardiovascular microcirculation (13), mitochondrial dysfunction, inflammation, and oxidative stress (14, 15). Exercise also counteracts age-related adverse body composition changes, preserving muscle mass, reducing visceral fat, and enhancing strength, bone density, and mobility, collectively lowering cardiovascular and all-cause mortality.

Current strategies for reducing epicardial adipose tissue (EAT) include pharmacological therapy, dietary or other weight-loss interventions, bariatric surgery, and structured exercise. Cardiometabolic agents such as glucagon-like peptide-1 receptor agonists, sodium-glucose cotransporter-2 inhibitors, thiazolidinediones, dipeptidyl peptidase-4 inhibitors, and statins have shown EAT-lowering effects, particularly in patients with obesity or type 2 diabetes (16, 17). Diet-induced weight loss and bariatric surgery can also reduce EAT, but their feasibility, invasiveness, long-term adherence, and applicability to generally healthy community-dwelling older adults may be limited (16). Exercise is therefore especially attractive in older populations because it is non-pharmacological, scalable, and can simultaneously improve cardiorespiratory fitness, physical function, muscle health, metabolic risk, and independence. Meta-analyses of randomized trials indicate that exercise alone can reduce EAT even without a concomitant weight-loss intervention (18, 19). However, most available exercise trials have involved younger or metabolically diseased populations, lasted only a few weeks to several months, and have provided limited evidence regarding long-term exercise frequency-response relationships in community-dwelling older adults.

However, the extent to which a structured, long-term exercise program can reduce EAV in community-dwelling older adults remains unclear, and whether changes in pro-inflammatory factors accompany such reductions has not been thoroughly examined. Therefore, this study was designed to evaluate the effect of a 12-month structured exercise intervention on EAV and to explore concomitant changes in circulating pro-inflammatory cytokines, with particular attention to the dose–response relationship between weekly exercise frequency and EAV reduction.

## 2 Methods

### 2.1 Study design

This was a single-center, pragmatic randomized controlled trial conducted at Fujian Provincial Hospital. The study used a post-randomization consent (Zelen) design. We selected this approach because a year-long behavioral intervention requires substantial participant motivation and repeated attendance; informing all participants about the alternative structured exercise program before allocation could increase disappointment, behavioral contamination, or differential adherence among those assigned to usual care. Zelen designs have been used in pragmatic and behavioral trials to reduce such disappointment/Hawthorne effects and to estimate the real-world effect of offering an intervention under conditions of incomplete uptake (20–22). In the present study, randomization therefore preceded consent to the assigned strategy, while all participants provided written informed consent before any study-specific baseline assessment or intervention procedure. We recognize that post-randomization refusal can reduce statistical efficiency and introduce selection bias. Because this was an exploratory trial, no formal sample-size calculation or inflation for anticipated post-randomization refusal was performed. Of 156 randomized individuals, 13 (8.3%) declined post-randomization consent; 143 entered follow-up and 118 completed the 12-month assessment. These features are considered when interpreting the findings and are explicitly acknowledged as limitations.

The study protocol was approved by the Ethics Committee of Fujian Provincial Hospital (Approval No. K2021-12-076) and was conducted in accordance with the principles of the Declaration of Helsinki. Written informed consent was obtained from all participants before study procedures.

### 2.2 Participants

Recruitment used hospital posters, WeChat advertisements, and physician referrals; some participants were recruited from clinical care. Interested individuals were screened by geriatricians.

We prospectively recruited community-dwelling older adults aged ≥ 60 years who were functionally independent and able to complete a 400-m walk within 15 min without assistance and who voluntarily agreed to participate in the 12-month exercise program and follow-up. Participants were excluded if they had any absolute or relative contraindication to exercise training or cardiopulmonary assessment, including: (1) acute myocardial infarction (within 3–5 days), unstable angina, or resting ECG suggesting acute ischemia; (2) active endocarditis, myocarditis, or pericarditis; (3) uncontrolled symptomatic arrhythmias or high-grade atrioventricular block without a pacemaker; (4) acute heart failure or NYHA class III–IV; (5) aortic dissection, aneurysm, or severe valvular disease; (6) uncontrolled asthma, pulmonary oedema, significant pulmonary hypertension, or resting oxygen saturation ≤ 85%; (7) other severe uncontrolled diseases (e.g., hypertension, diabetes, thyrotoxicosis, severe renal insufficiency, infection); (8) moderate–severe cognitive impairment (MMSE ≤ 20) or mental disorder precluding cooperation; (9) atrial fibrillation, frequent premature beats, or metallic implants interfering with cardiac MRI; (10) communication difficulties; or (11) inability or unwillingness to provide informed consent.

Relevant medication exposure was not used as an automatic exclusion criterion unless the associated condition was severe or uncontrolled and therefore constituted an exercise contraindication. Use of antihypertensive agents, antidiabetic drugs, and statins was systematically recorded at baseline, together with medical history. These medication variables were compared between groups to assess potential pharmacological confounding; no significant baseline between-group differences were observed (Table 1).

**Table 1:** Demographic and geriatric characteristics of participants at baseline.

Characteristics	Total (*N* = 118)	CON (*n* = 57)	STE (*n* = 61)	*p*-value
Age, years	69.17 ± 4.41	68.56 ± 4.31	69.74 ± 4.45	0.148
BMI	23.58 ± 2.66	23.87 ± 2.86	23.31 ± 2.46	0.251
Waistline, cm	87.97 ± 8.31	88.64 ± 9.01	87.35 ± 7.62	0.404
Waist-Hip Ratio	0.91 ± 0.06	0.91 ± 0.06	0.92 ± 0.07	0.361
Systolic BP, mmHg	131.42 ± 18.26	134.54 ± 19.29	128.49 ± 16.87	0.072
Diastolic BP, mmHg	72.50(66.75, 83.00)	72.00(67.00, 83.50)	74.00(66.00, 83.00)	0.861
MMSE	28.20 ± 1.46	27.95 ± 1.37	28.44 ± 1.51	0.069
6MWT, m	470.60 ± 55.65	464.54 ± 55.15	476.08 ± 55.97	0.262
Gait speed, m/s	1.42 ± 0.24	1.39 ± 0.24	1.44 ± 0.24	0.272
MGS, kg	26.78 ± 9.27	25.56 ± 9.58	27.89 ± 8.91	0.175
5STS, s	8.24 ± 1.44	8.34 ± 1.46	8.15 ± 1.43	0.458
PA, MET-min/week	3577.46 ± 1695.64	3451.42 ± 1819.15	3695.23 ± 1577.59	0.437
Triglyceride, mmol/L	1.39 (0.94, 1.83)	1.39 (0.99, 1.98)	1.38 (0.93, 1.79)	0.592
Cholesterol, mmol/L	4.86 ± 1.22	4.86 ± 1.17	4.87 ± 1.27	0.981
LDL-C, mmol/L	3.16 ± 0.95	3.12 ± 0.92	3.20 ± 0.98	0.643
HDL-C, mmol/L	1.32 ± 0.32	1.37 ± 0.31	1.28 ± 0.33	0.153
FPG, mmol/L	5.75(5.23, 6.43)	5.75 (5.33, 6.47)	5.75 (5.21, 6.40)	0.537
HbA1c, %	6.00 (5.70, 6.43)	6.00 (5.65, 6.45)	6.00 (5.70, 6.45)	0.483
BFM, kg	18.79 ± 5.22	19.87 ± 5.40	17.79 ± 4.87	**0.030**
PBF, %	30.01 ± 7.13	32.05 ± 7.00	28.10 ± 6.75	**0.002**
VFA, cm^2^	90.52 ± 30.15	97.85 ± 32.08	83.66 ± 26.71	**0.010**
EAV, mL	83.73 (61.82, 112.75)	81.06 (59.69, 113.24)	86.53 (63.17, 110.71)	0.550
Male	55(46.6)	29(50.9)	26(42.6)	0.369
Hypertension	63(53.4)	32(53.3)	31(53.4)	0.990
Diabetes	39(33.1)	18(31.6)	21(34.4)	0.742
Coronary heart disease	7(5.9)	3(5.0)	4(6.9)	0.963
Smoking	19(16.1)	9(15.8)	10(16.4)	0.929
Alcohol	6(5.1)	4(6.7)	2(3.4)	0.707
Antihypertensive agent	64(54.2)	31(54.4)	33(54.1)	0.975
Antidiabetic drug	30(25.4)	12(21.1)	18(29.5)	0.292
Statin	47(39.8)	20(35.1)	27(44.3)	0.309

BMI, body mass index; MGS, maximal grip strength; MMSE, Mini-mental state examination; 6MWT, 6-min walking test; 5STS, 5-times sit-to-stand test; PA, physical activities; MET, metabolic equivalent; LDL-C, low-density lipoprotein cholesterol; HDL-C, high-density lipoprotein cholesterol; FPG, fasting plasma glucose; HbA1c, glycated hemoglobin. BFM, body fat mass; PBF, percent body fat; VFA, visceral fat area; EAV, epicardial adipose volume. Data are presented as mean ± SD or n (%); bold type indicates a *P*-value < 0.05.

#### 2.2.1 General data collection

Baseline demographic characteristics, socioeconomic information, medical history, medication use, lifestyle factors, and previous disease history were collected through standardized face-to-face interviews conducted by trained researchers.

Trained researchers collected demographic and sociological data (name, sex, age, marital status, residence), medical history (coronary disease, hypertension, type 2 diabetes, other significant conditions, falls), and lifestyle factors (smoking, alcohol) via face-to-face questionnaires.

#### 2.2.2 Physical fitness and functional assessment

We measured height, weight, BMI, blood pressure, heart rate, gait speed, 6-min walk distance (23), five-times sit-to-stand time (24), and grip strength (25) using standardized methods. Body fat mass, percent body fat, visceral fat area, and waist-to-hip ratio were assessed by bioelectrical impedance analysis. Daily physical activity and weekly energy expenditure (MET-min/week) were estimated using the International Physical Activity Questionnaire (IPAQ) (26).

#### 2.2.3 Laboratory tests

Fasting morning blood samples were analyzed for triglycerides, HDL-C, LDL-C, glucose, HbA1c, IL-1R, IL-2R, IL-1β, IL-18, MCP-1, and M-CSF. Serum concentrations of inflammatory biomarkers were quantified using the Bio-Plex Pro Human Cytokine 48-Plex Panel (Bio-Rad Laboratories, Hercules, CA, United States; Cat. No. 12007283) on a Bio-Plex 200 system with Luminex xMAP technology, according to the manufacturer’s instructions. Briefly, serum samples were incubated with magnetic capture beads, followed by biotinylated detection antibodies and streptavidin-phycoerythrin, and fluorescence signals were measured on the Bio-Plex 200 system. The manufacturer specifies intra-assay precision of ≤15% coefficient of variation (CV) and inter-assay precision of ≤25% CV for this panel. Because analytical sensitivity is analyte- and lot-dependent, the lower detection/quantification boundary for each cytokine was evaluated against its assay-specific standard curve; values outside the validated working range were not used for quantitative interpretation. All samples were processed under standardized laboratory conditions with quality-control procedures applied throughout the assay.

#### 2.2.4 Cardiac magnetic resonance imaging and image analysis

Cardiac MRI was performed on a 3.0 T Siemens Prisma scanner using balanced steady-state free precession cine sequences (short-axis and long-axis; TR/TE 39.24/1.43 ms, FOV 340 × 282 mm, flip angle 80°). EAV was quantified with CVi42 software (Circle Cardiovascular Imaging) by manually tracing fat areas on end-diastolic short-axis slices and multiplying by slice thickness (Figure 1). A blinded radiologist performed all analyses; the method has been validated for reproducibility (27, 28).

**Figure 1:**
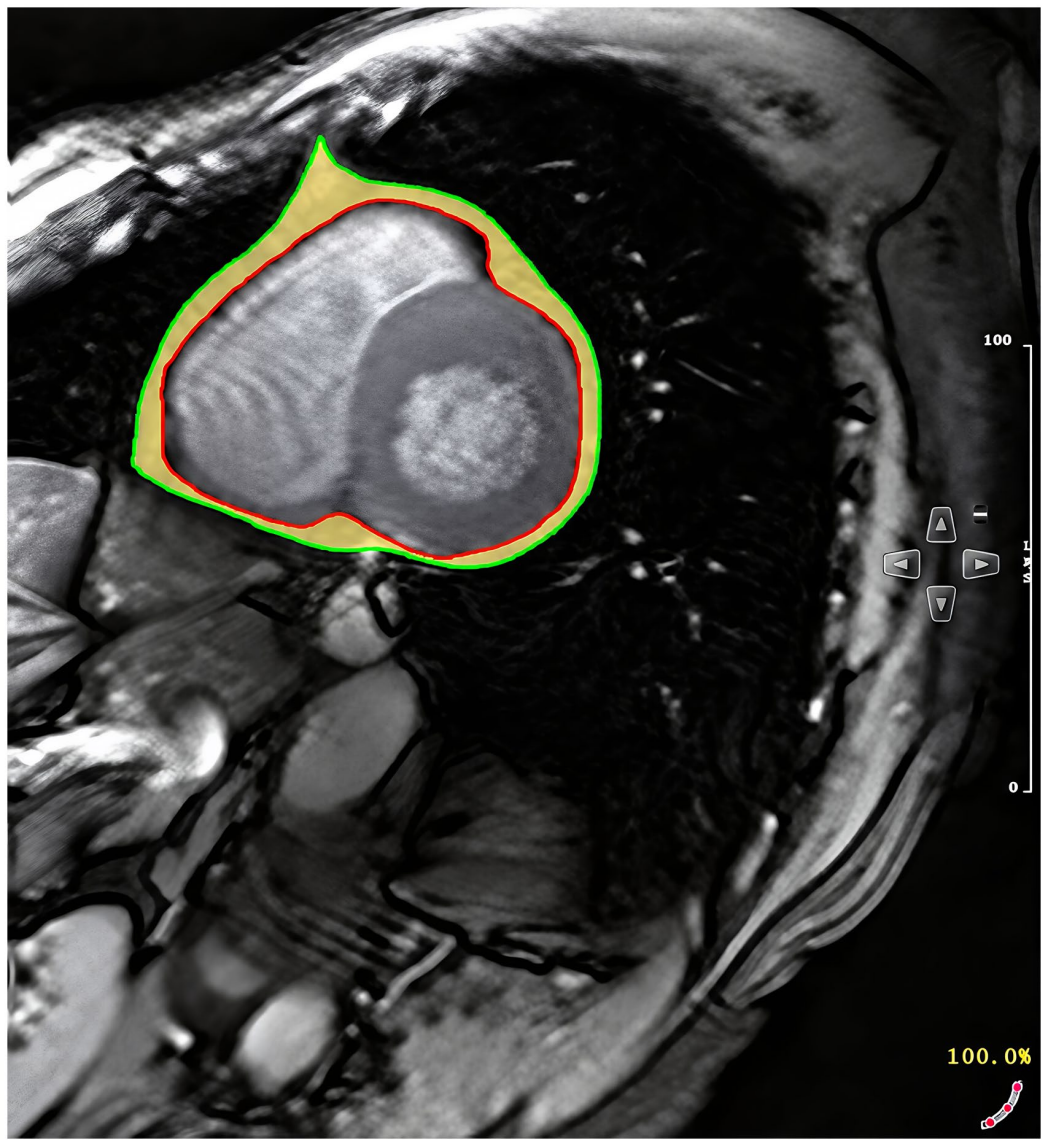
Schematic illustration of CMR post-processing for quantifying epicardial adipose tissue volume.

### 2.3 Randomization and allocation

Eligible participants were randomly allocated 1:1 using a computer-generated sequence. Those assigned to STE were informed of the intervention and follow-up; if they agreed and gave written consent, they underwent baseline assessment. Control participants were told about the follow-up but not about the other group; those consenting were enrolled. Baseline assessments and intervention began within 2 weeks. Outcome assessors, data monitors, and statisticians were blinded to group assignment.

### 2.4 Intervention (STE group)

Individualized exercise programs were developed by geriatric and rehabilitation physicians. Participants attended one supervised center-based session weekly and performed at least two home-based sessions of a similar modality each week for 12 months. In the first four center sessions, supervisors taught correct techniques and provided equipment and exercise charts.

Aerobic exercise: initial intensity at the anaerobic threshold; duration increased from 15–20 min to 35 min per session. Center-based aerobic training used a treadmill or cycle ergometry, with heart rate, blood pressure, and ECG monitoring. Each session included 5–10 min of warm-up and cool-down.

Resistance exercise: performed 2–3 sessions/week using elastic bands, dumbbells, or wrist weights. The initial load was 30–40% of estimated 1-RM for the upper body and 50–60% for the lower body. Seven major muscle groups were targeted (upper limbs, shoulders, chest, back, abdomen, glutes, and lower limbs), with 1–2 sets of 8–12 repetitions per group; resistance was progressively increased.

Flexibility and balance: stretching of the seven muscle groups 2–3 sessions/week; balance exercises (heel-to-toe walking, tandem walking, single-leg stance, squats) 1–3 sessions/week.

Participants kept exercise diaries recording frequency and perceived exertion. Supervisors reviewed diaries weekly, encouraged adherence, and adjusted intensity as needed. Intervention adherence was defined as completing ≥ 75% of prescribed sessions over the year.

### 2.5 Control group

After four initial education sessions, controls were encouraged (but not required) to engage in self-directed exercise (walking, cycling, swimming, dancing, table tennis) and were invited to educational salons, but had no trainer contact or structured training. Monthly telephone interviews monitored health and spontaneous physical activity.

### 2.6 Outcomes

Primary outcome: absolute change in EAV from baseline to 12 months. Secondary outcomes: absolute changes in body fat parameters (fat mass, percent fat, visceral fat area) and serum pro-inflammatory cytokines (IL-1R, IL-2R, IL-1β, IL-18, MCP-1, M-CSF).

### 2.7 Statistical analysis

No sample size calculation was performed because this was an exploratory study. Graphs were created with GraphPad Prism 8.0; analyses were performed using SPSS 26.0. *p* < 0.05 was considered significant. Analyses included only participants with complete baseline and follow-up data. Continuous variables are reported as mean ± SD; normality and homogeneity were checked with the Shapiro–Wilk and Levene’s tests. Between-group baseline comparisons used independent *t*-tests, Mann–Whitney *U*, or chi-square/Fisher’s exact tests as appropriate. Within-group changes were tested with paired *t*-tests or Wilcoxon signed-rank tests. Between-group differences in absolute changes were tested with independent *t*-tests or Mann–Whitney *U*; 95% CIs were estimated with Hodges–Lehmann. ANCOVA adjusted for baseline values was used to compare changes between groups. Univariate linear regression examined the association between average weekly exercise frequency and EAV change; multivariate linear regression explored factors influencing EAV change, adjusting for baseline age, BMI, waist circumference, total cholesterol, and HbA1c.

## 3 Results

### 3.1 Participants

Of 174 individuals recruited, 18 were ineligible and 156 underwent randomization (78 per group). After allocation, 8 participants assigned to the control group and 5 assigned to STE declined post-randomization consent, leaving 70 controls and 73 STE participants who entered follow-up. During the 12-month study, 13 controls and 12 STE participants withdrew; consequently, 57 controls and 61 STE participants completed the trial and were included in the complete-case analysis (Figure 2). Baseline characteristics are shown in Table 1, and baseline cytokine levels in Table 2. Of the 118 completers, 55 (46.6%) were men, with a mean age of 69.17 ± 4.41 years. There were no significant between-group differences at baseline in EAV, cytokines, demographics, lifestyle, chronic diseases, antihypertensive agents, antidiabetic drugs, statin use, or physical fitness. Body fat mass, percent body fat, and visceral fat area differed at baseline and were therefore addressed using baseline-adjusted analyses (Table 3).

**Figure 2:**
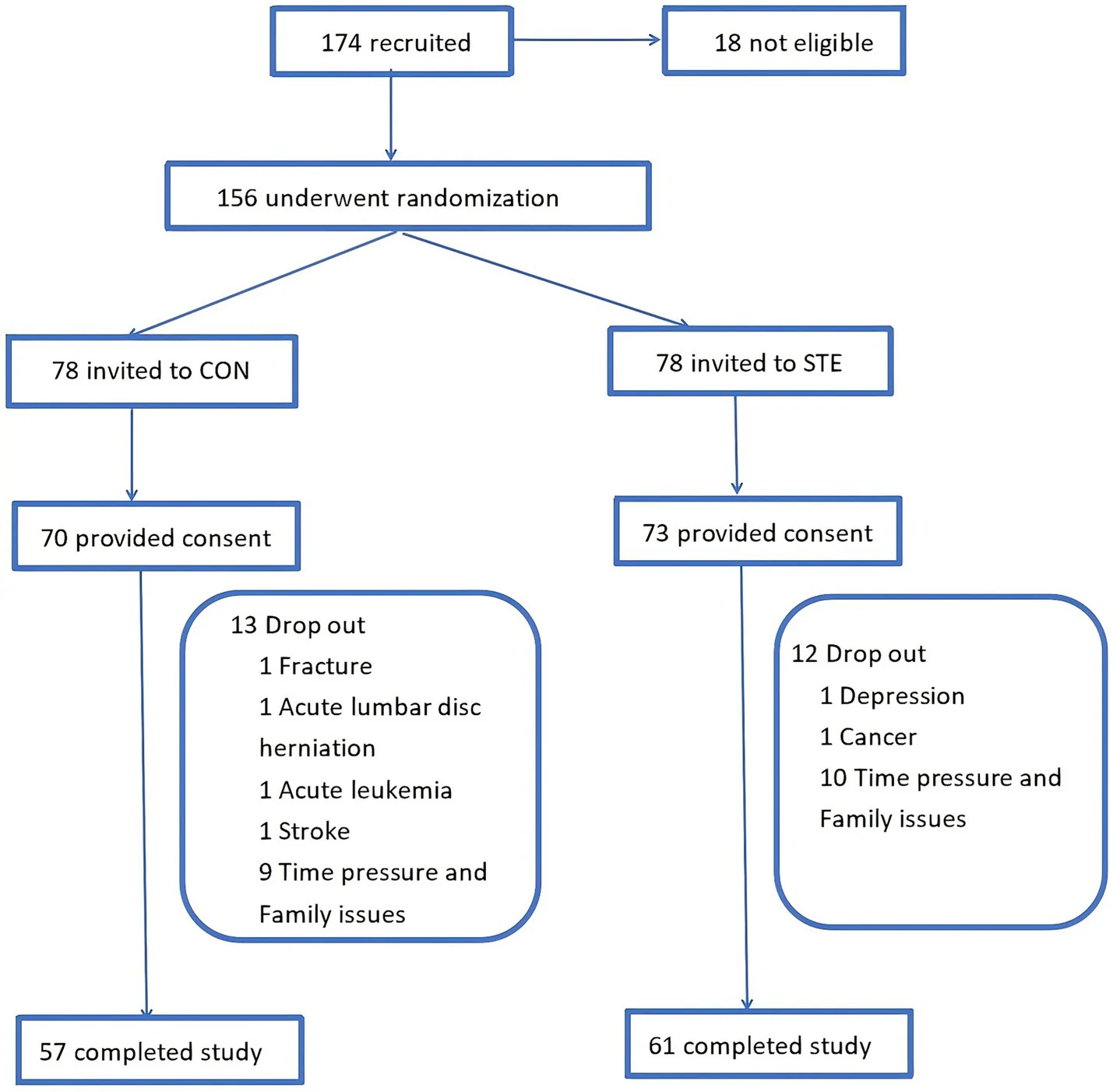
Research flow chart. CON, control group; STE, structured exercise group.

**Table 2:** Serum cytokines of participants at baseline.

Characteristics	Total (*N* = 112)	CON (*n* = 55)	STE (*n* = 57)	*p*-value
IL-1R, pg/mL	74.35(45.54, 100.20)	74.35(45.54, 101.70)	74.35(64.69, 98.70)	0.958
IL-2R, pg/mL	34.07(25.58, 46.37)	35.14(25.18, 50.82)	33.63(26.72, 45.58)	0.775
IL-1β, pg/mL	0.96(0.68, 1.27)	0.92(0.68, 1.27)	1.00(0.68, 1.27)	0.724
IL-18, pg/mL	6.06(2.83, 11.73)	7.72(2.94, 13.52)	5.66(2.64, 10.22)	0.267
MCP-1, pg/mL	6.74(4.40, 9.61)	6.47(4.74, 9.52)	6.74(3.83, 11.05)	0.981
M-CSF, pg/mL	8.65(6.52, 12.04)	8.72(6.52, 12.32)	8.58(6.04, 11.34)	0.602
IL-4, pg/mL	2.65(1.95, 3.44)	2.54(1.95, 2.99)	2.76(1.86, 3.53)	0.614
IL-13, pg/mL	0.42(0.35, 0.70)	0.42(0.35, 0.81)	0.57(0.35, 0.70)	0.948

IL-1R, interleukin-1 receptor; IL-2R, interleukin-2 receptor; IL-1β, interleukin-1β; IL-18, interleukin-18; MCP-1, monocyte chemotactic protein-1; M-CSF, macrophage colony stimulating factor; IL-4, interleukin −4; IL-13, interleukin-13.

**Table 3:** Change of EAV, adiposity indicators and the inter-group differences.

Characteristics	Absolute changes		Difference in absolute change (95%CI)	Difference, *p*-value	Adjusted difference (95% CI)	Adjusted difference, *p*-value
	CON (*n* = 57)	STE (*n* = 61)				
EAV, mL	−2.61 ± 22.37	−17.76 ± 18.48	−15.15(−22.61, -7.69)	**<0.001**	−14.29(−20.29, −8.29)	**<0.001**
BFM, kg	−1.02 ± 1.65	−0.87 ± 1.90	0.15(−0.50, 0.80)	0.648	0.06(−0.58, 0.70)	0.852
PBF, %	−1.38 ± 2.18	−1.11 ± 2.39	0.27(−0.57, 1.10)	0.527	0.02(−0.84, 0.87)	0.972
VFA,cm^2^	−5.83 ± 9.54	−4.15 ± 11.80	1.68(−2.25, 5.61)	0.4	0.16(−3.64, 3.96)	0.935

EAV, epicardial adipose volume; BFM, body fat mass; PBF, percent body fat; VFA, visceral fat area. Data were adjusted by age and baseline data. Bold type indicates a *P*-value < 0.05.

### 3.2 Correlation between EAV and pro-inflammatory factors

Spearman correlation showed EAV significantly positively correlated with IL-1R, IL-2R, IL-18, MCP-1, and M-CSF (*r* = 0.204, 0.251, 0.196, 0.214, and 0.243, respectively). After log transformation, EAV also correlated with IL-1β (*r* = 0.194) (Figure 3).

**Figure 3:**
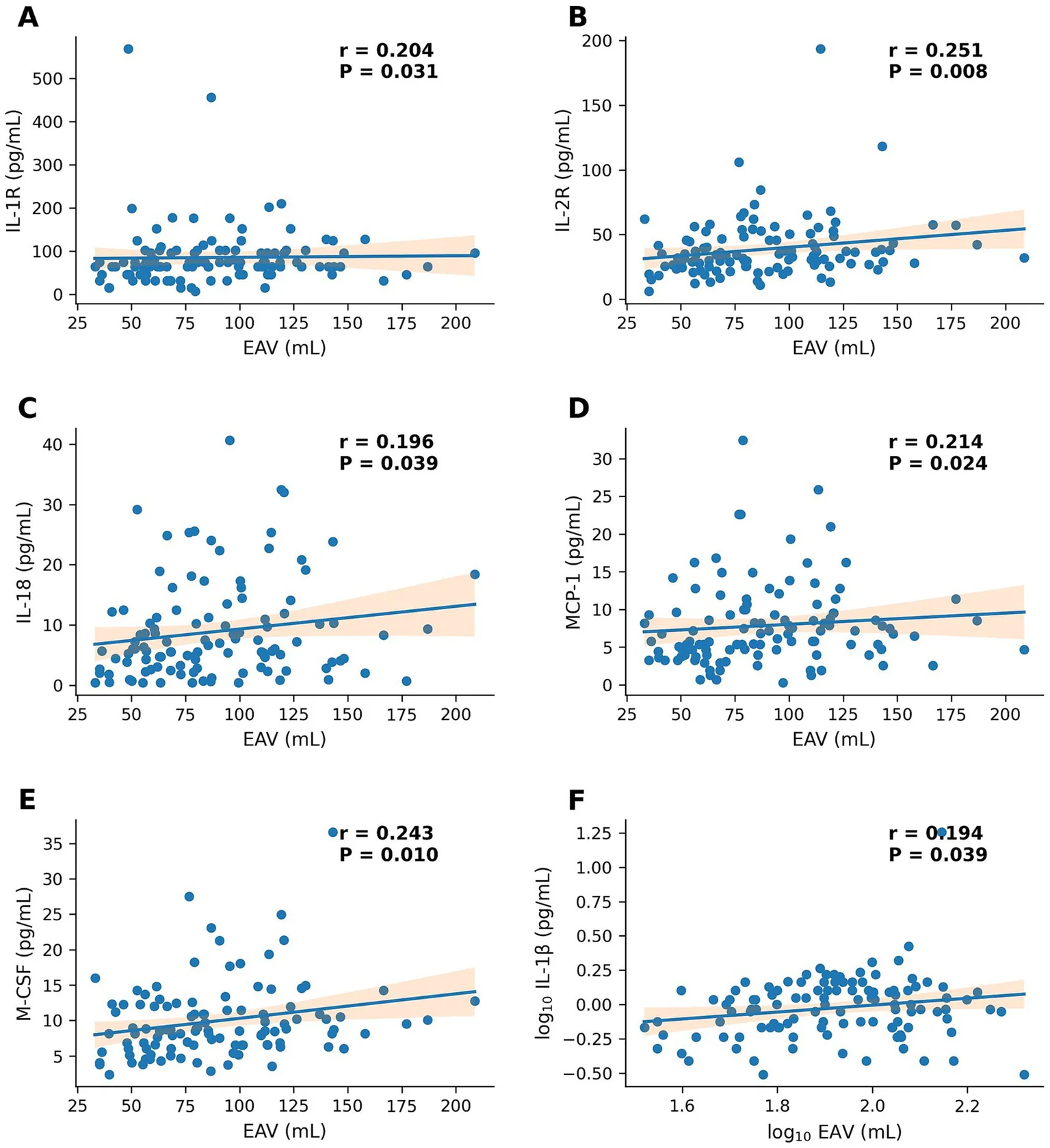
Associations between epicardial adipose volume (EAV) and serum pro-inflammatory factors. Scatterplots show the associations of EAV with **(A)** IL-1R, **(B)** IL-2R, **(C)** IL-18, **(D)** MCP-1, **(E)** M-CSF, and **(F)** IL-1β. Solid lines represent fitted linear trends, and shaded areas indicate 95% confidence intervals. Spearman rank correlation coefficients (*r*) and corresponding *p* values are reported for IL-1R, IL-2R, IL-18, MCP-1, and M-CSF. For panel **(F)**, EAV and IL-1β were log10-transformed, and Pearson correlation analysis was performed. EAV, epicardial adipose volume; IL-1R, interleukin-1 receptor; IL-2R, interleukin-2 receptor; IL-18, interleukin-18; MCP-1, monocyte chemoattractant protein-1; M-CSF, macrophage colony-stimulating factor; IL-1β, interleukin-1β.

### 3.3 Effect of STE on EAV and adiposity indices

The absolute EAV change differed significantly between groups: STE –17.66 ± 18.48 mL vs. control −2.61 ± 22.37 mL; mean difference −15.15 mL (95% CI –22.61 to −7.69; *p* < 0.0001). This remained significant after adjustment for baseline EAV and age (adjusted difference −14.29 mL, *p* < 0.001; Table 3).

In contrast, changes in body fat mass (adjusted mean difference 0.06 kg, 95% CI –0.58 to 0.70), percent body fat (0.02%, −0.84 to 0.87), and visceral fat area (0.16 cm^2^, −3.64 to 3.96) were not significant between groups (all *p* > 0.05; Table 3).

### 3.4 Effect of STE on pro-inflammatory factors

Absolute changes in cytokines are presented in Table 4. Unadjusted between-group differences were significant for IL-2R (−13.14 pg/mL, 95% CI –26.07 to −0.22; *p* = 0.046) and MCP-1 (−2.29 pg/mL, −4.28 to −0.31; *p* = 0.024). However, after adjusting for baseline levels, only M-CSF showed a significant difference: −1.75 pg/mL (95% CI –3.34 to −0.16; *p* = 0.032) (Table 4).

**Table 4:** Change of pro-inflammatory factors and the inter-group differences.

Characteristics	Absolute changes		Difference in absolute change (95%*CI*)	Difference, *p*-value	Adjusted difference (95%CI)	Adjusted difference, *p*-value
	CON (*n* = 41)	STE (*n* = 42)				
IL-1R, pg/mL	−55.98 ± 71.81	−53.44 ± 50.78	2.54(−24.57, 29.65)	0.852	–	*–*
IL-2R, pg/mL	4.34 ± 40.35	−8.81 ± 11.91	−13.14(−26.07, −0.22)	**0.046**	−10.27(−21.35, 0.80)	0.069
IL-1β, pg/mL	−0.26 ± 0.44	−0.76 ± 2.31	−0.50(−1.23, 0.23)	0.178	–	–
IL-18, pg/mL	−1.90 ± 7.52	−3.70 ± 5.55	−1.81(−4.70, 1.09)	0.218	–	–
MCP-1, pg/mL	−3.60 ± 4.06	−5.89 ± 4.97	−2.29(−4.28, -0.31)	**0.024**	−0.93(−1.96, 0.12)	0.08
M-CSF, pg/mL	−0.89 ± 3.86	−2.49 ± 3.93	−1.60(−3.30, 0.10)	**0.064**	−1.75(−3.34, −0.16)	**0.032**

IL-1R, interleukin −1 receptor; IL-2R, interleukin −2 receptor; IL-1β, interleukin 1β; IL-18, interleukin-18; MCP-1, monocyte chemotactic protein 1; M-CSF, macrophage colony stimulating factor. Bold values indicate statistical significance at *p* < 0.05.

### 3.5 Dose–response relationship between exercise frequency and EAV change

Univariate linear regression using average weekly exercise frequency as the independent variable yielded an *R*^2^ of 0.084 and a coefficient of −3.156 (95% CI –5.070 to −1.242), meaning each additional session per week predicted a 3.156 mL greater EAV reduction (Figure 4).

**Figure 4:**
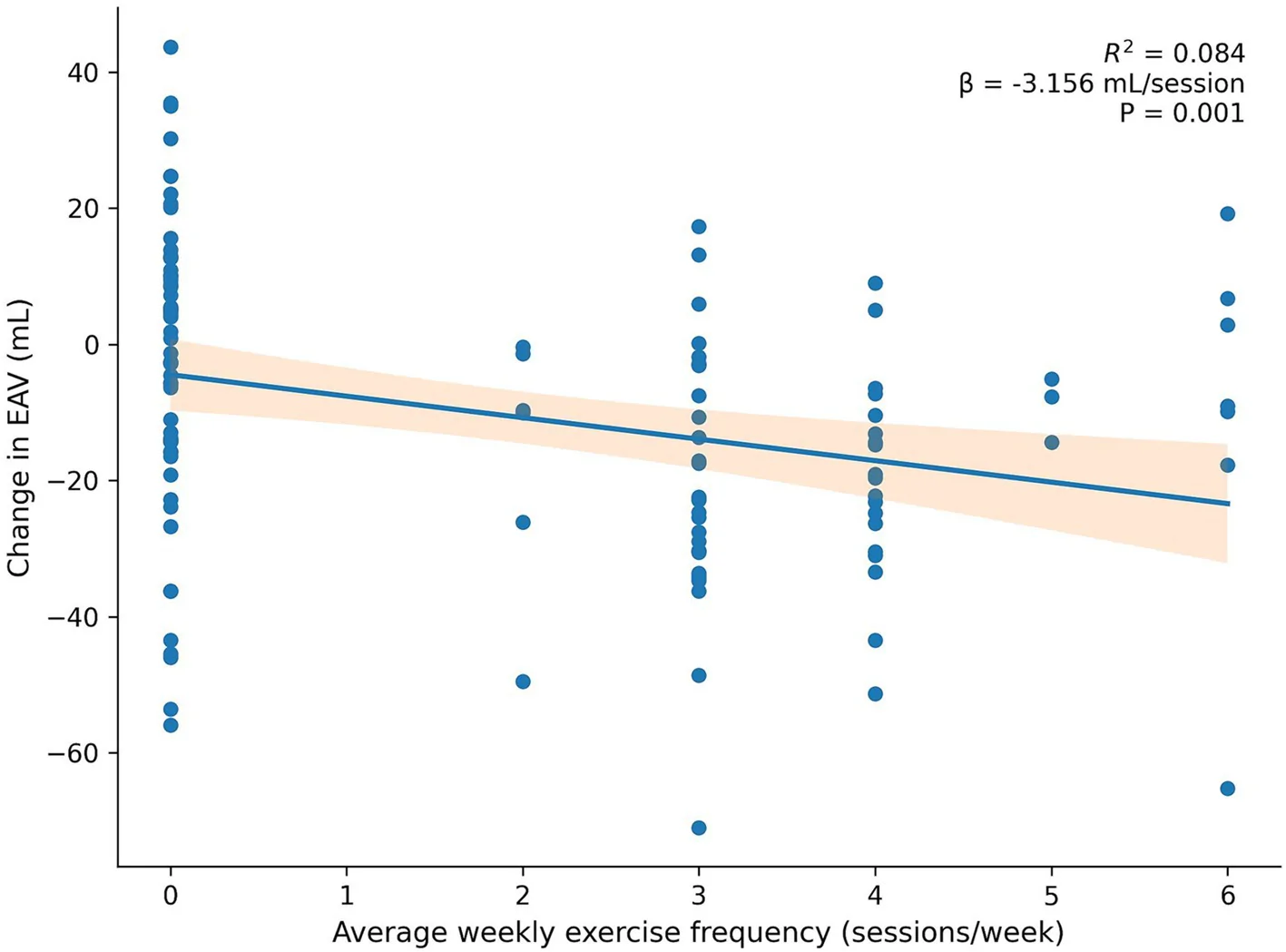
Association between average weekly exercise frequency and change in epicardial adipose volume (EAV). Each point represents an individual participant. The solid line represents the fitted linear regression, and the shaded area indicates the 95% confidence interval. Univariate linear regression showed that each additional weekly exercise session was associated with a 3.156 mL greater reduction in EAV (*β* = −3.156 mL/session, 95% CI: −5.070 to −1.242; *R2* = 0.084; *p* = 0.001). EAV, epicardial adipose volume.

In multivariate analysis (*R*^2^ = 0.481, adjusted *R*^2^ = 0.443), after adjusting for baseline age, BMI, waist circumference, total cholesterol, and HbA1c, the independent predictors of greater EAV reduction were: higher weekly exercise frequency (*β* = −2.891, *p* < 0.001), higher baseline EAV (*β* = −0.340, *p* < 0.001), and lower baseline triglycerides (*β* = 4.846, *p* = 0.021) (Table 5).

**Table 5:** Multiple linear regression of absolute changes of epicardial adipose volume.

Variable	*β*	SE	Standardized *β*	*p*-value
Exercise frequency, sessions/week	−2.891	0.782	−0.266	**<0.001**
Baseline EAV, mL	−0.340	0.043	−0.560	**<0.001**
Baseline TG, mmol/L	4.846	2.076	0.176	**0.021**

EAV, epicardial adipose volume. TG, triglycerides. Bold values indicate statistical significance at *p* < 0.05.

## 4 Discussion

This study demonstrates that a 12-month structured exercise program significantly reduces EAV in community-dwelling older adults, and the reduction exhibits a clear frequency-dependent (dose–response) pattern. Moreover, the intervention lowered serum M-CSF, one of several pro-inflammatory cytokines examined, although other classical inflammatory markers did not change significantly after adjustment. Our findings reinforce the value of regular exercise as a non-pharmacological approach to reduce cardiac adiposity and modulate inflammation in aging populations.

We observed statistically significant but generally weak positive correlations between EAV and several circulating pro-inflammatory factors (IL-1R, IL-2R, IL-18, MCP-1, M-CSF, and IL-1β), consistent with previous reports linking EAT with systemic inflammatory activity (29). However, the correlation coefficients were modest (approximately *r* = 0.19–0.25), and the scatterplots, especially those for IL-18 and MCP-1, showed substantial dispersion. Accordingly, these analyses should be regarded as exploratory and do not establish a direct or causal relationship between EAV and individual cytokines. EAT is metabolically and immunologically active and can participate in local and systemic inflammatory signaling (7, 29); nevertheless, circulating cytokine concentrations are influenced by multiple tissues and physiological factors.

The observed EAV reduction of ~15 mL (or ~14 mL after adjustment) in the exercise group is clinically meaningful. Prior randomized trials have reported EAT reductions after exercise, but most were shorter (3–6 months) and often combined with dietary restriction. For instance, Christensen et al. (30) found that 3 months of resistance or endurance training reduced epicardial fat mass by 24–32% in abdominal obesity patients. Fernandez-del-Valle et al. (31) reported EAT reduction after only 3 weeks of high-intensity resistance training in obese women. In contrast, Jonker et al. (32) found no EAT change after 6 months of unsupervised moderate exercise in diabetic patients. Our longer, partially supervised program with a higher weekly volume likely accounts for the positive effect, supporting the need for sufficient intensity and supervision in older populations.

Notably, while the exercise group showed a significant EAV reduction, we did not observe significant changes in general adiposity indices (body fat mass, percent fat, or visceral fat area) after baseline adjustment. This pattern is consistent with meta-analytic evidence showing that exercise can reduce EAT even when changes in body weight or BMI are small or absent (18, 19). A plausible explanation is the distinctive lipid metabolism of EAT: compared with other fat depots, epicardial fat has been reported to have a greater capacity for free-fatty-acid uptake and release and relatively high rates of fatty-acid synthesis and breakdown, supporting rapid lipid turnover. Exercise may further promote mobilization of cardiac fat through increased metabolic demand and lipolytic signaling. These physiological characteristics may help explain why EAV can decrease despite limited measurable changes in broader adiposity indices. Nevertheless, because our study did not directly measure EAT lipolysis, blood flow, or tissue metabolism, this mechanism remains an interpretation rather than a demonstrated causal pathway (18, 33).

Regarding inflammation, only M-CSF remained significantly lower in the exercise group after adjustment. M-CSF is an important regulator of monocyte/macrophage survival, differentiation, and tissue accumulation, and macrophage-rich adipose tissue is a major contributor to obesity-related low-grade inflammation. Exercise and lifestyle interventions can reduce adipose-tissue macrophage infiltration and inflammatory signaling (34, 35). We therefore speculate that the observed decrease in circulating M-CSF may reflect reduced monocyte/macrophage stimulation accompanying exercise-induced remodeling of metabolically active adipose depots, including EAT. In parallel, repeated exercise may reduce EAV through cumulative activation of lipolytic pathways, increased fatty-acid oxidation, improved insulin sensitivity, and mitochondrial adaptations (36, 37). These processes provide a biologically plausible explanation for the observed association between higher weekly exercise frequency and greater EAV reduction. Importantly, circulating M-CSF was measured as a systemic biomarker and no EAT biopsy, macrophage phenotyping, or mediation analysis was performed; thus, our data do not establish that M-CSF mediates EAV loss.

Our dose–response analysis provides novel evidence that EAV reduction is frequency-dependent. Each additional weekly exercise session was associated with an approximately 3 mL greater decrease in EAV, independent of other factors. This finding has practical implications: clinicians can encourage older adults to increase exercise frequency rather than only intensity or duration. While high-intensity interval training (HIIT) has been reported to be superior to moderate-intensity continuous training (24, 27, 28), older adults may not tolerate HIIT well. Our data suggest that simply increasing the number of moderate-intensity sessions per week can yield meaningful EAV reduction, offering a feasible and safe strategy.

We also found that higher baseline EAV and lower baseline triglycerides predicted greater EAV reduction. This suggests that individuals with more cardiac fat and a healthier lipid profile may benefit most from exercise. Combining exercise with dietary modification to lower triglycerides could potentially enhance the effect, a hypothesis that warrants further investigation.

### 4.1 Strengths

Our study recruited from the community and used a partially supervised, long-term exercise protocol with good adherence. We used cardiac MRI, the gold standard for EAV quantification, with semi-automated software showing excellent reproducibility. The Zelen design allowed a more realistic assessment of real-world acceptance and adherence. The control group received health education and standard guidance, reflecting ethical practice and highlighting the added benefit of supervised training.

### 4.2 Limitations

First, participants were volunteers, which may introduce selection bias and limit generalizability. This concern is particularly relevant to the post-randomization consent (Zelen) design: 13 of 156 randomized individuals declined consent after allocation, and an additional 25 participants withdrew during follow-up. No formal sample-size calculation or inflation for refusal/crossover was performed because the trial was exploratory, and the complete-case analysis may therefore be vulnerable to attrition-related bias and reduced statistical power. Second, although medication use (including antihypertensive, antidiabetic, and statin therapy) and multiple baseline covariates were recorded and compared between groups, residual confounding from medication changes, diet, and other lifestyle factors cannot be excluded. Third, the sample size was modest, limiting power to detect differences in secondary inflammatory markers and adiposity indices. The lack of significant changes in other cytokines may be due to type II error. Fourth, we did not perform formal mediation analysis; therefore, we cannot conclude that M-CSF mediates the EAV reduction. Our title and discussion have been carefully worded to reflect this. Future studies with larger samples and mechanistic designs (e.g., causal mediation models) are needed to clarify the pathway.

### 4.3 Conclusion and implications

A 12-month structured exercise program effectively reduced EAV in community-dwelling older adults in a frequency-dependent manner and concurrently lowered serum M-CSF. These results suggest that regular, supervised exercise, even of moderate intensity, can be a practical strategy to reduce cardiac fat and modulate inflammation in aging populations. The dose–response relationship supports recommending a higher weekly frequency of exercise sessions. Further research is required to establish whether this EAV reduction translates into improved cardiovascular outcomes and to identify the exact molecular mediators.

## Data Availability

The raw data supporting the conclusions of this article will be made available by the authors, without undue reservation.
